# Prevalence of Genotypes That Determine Resistance of Staphylococci to Macrolides and Lincosamides in Serbia

**DOI:** 10.3389/fpubh.2017.00200

**Published:** 2017-08-28

**Authors:** Milena Mišić, Jelena Čukić, Dejan Vidanović, Milanko Šekler, Sanja Matić, Mihailo Vukašinović, Dejan Baskić

**Affiliations:** ^1^Department of Microbiology, Public Health Institute Vranje, Vranje, Serbia; ^2^Department of Clinical Microbiology, Laboratory for Virology, Serology, Immunology and Molecular Diagnostics, Public Health Institute Kragujevac, Kragujevac, Serbia; ^3^Department of Laboratory Diagnostics, National Reference Laboratory for Avian Influenza and Newcastle Disease of Poultry Republic of Serbia, Veterinary Specialized Institute Kraljevo, Kraljevo, Serbia; ^4^Faculty of Medical Sciences, Doctoral Studies, University of Kragujevac, Kragujevac, Serbia; ^5^Center for Molecular Medicine and Stem Cell Research, Faculty of Medical Sciences, University of Kragujevac, Kragujevac, Serbia

**Keywords:** staphylococci, MLS resistance, resistance phenotypes, *erm* genes, polymerase chain reaction genotyping, hospital-acquired infection, community-acquired infection

## Abstract

Macrolides, lincosamides, and streptogramins (MLS) resistance genes are responsible for resistance to these antibiotics in *Staphylococcus* infections. The purpose of the study was to analyze the distribution of the MLS resistance genes in community- and hospital-acquired *Staphylococcus* isolates. The MLS resistance phenotypes [constitutive resistance to macrolide–lincosamide–streptogramin B (cMLSb), inducible resistance to macrolide–lincosamide–streptogramin B (iMLSb), resistance to macrolide/macrolide–streptogramin B (M/MSb), and resistance to lincosamide–streptogramin A/streptogramin B (LSa/b)] were determined by double-disc diffusion method. The presence of the MLS resistance genes (*erm*A, *erm*B, *erm*C, *msr*A/B, *lnu*A, *lnu*B, and *lsa*A) were determined by end-point polymerase chain reaction in 179 isolates of staphylococci collected during 1-year period at the Center for Microbiology of Public Health Institute in Vranje. The most frequent MLS phenotype among staphylococcal isolates, both community-acquired and hospital-acquired, was iMLSb (33.4%). The second most frequent was M/MSb (17.6%) with statistically significantly higher number of hospital-acquired staphylococcal isolates (*p* < 0.05). MLS resistance was mostly determined by the presence of *msr*A/B (35.0%) and *erm*C (20.8%) genes. Examined phenotypes were mostly determined by the presence of one gene, especially by *msr*A/B (26.3%) and *erm*C (14.5%), but 15.6% was determined by a combination of two or more genes. M/MSb phenotype was the most frequently encoded by *msr*A/B (95.6%) gene, LSa/b phenotype by *lnu*A (56.3%) gene, and iMLSb phenotype by *erm*C (29.4%) and *erm*A (25.5%) genes. Although cMLSb phenotype was mostly determined by the presence of *erm*C (28.9%), combinations of two or more genes have been present too. This pattern was particularly recorded in methicillin-resistant *Staphylococcus aureus* (MRSA) (58.3%) and methicillin-resistant coagulase-negative staphylococci (MRCNS) (90.9%) isolates with cMLSB phenotype. The *msr*A/B gene and M/MSb phenotype were statistically significantly higher in hospital-acquired than community-acquired staphylococci strains (*p* < 0.05). There are no statistically significant differences between staphylococci harboring the rest of MLS resistance genes acquired in community and hospital settings (*p* > 0.05). The prevalence of iMLSb phenotypes may change over time, so it is necessary to perform periodic survey of MLS resistance phenotypes, particularly where the D-test is not performed routinely.

## Introduction

The *Staphylococcus* spp. includes at least 40 species, some of them may cause a wide variety of diseases in humans and animals, while some of them are generally non-pathogenic and considered to be commensal. *Staphylococcus aureus* is one of the most important bacteria that cause skin and soft tissue infections and number of serious other medical problems in human. Today, coagulase-negative staphylococci (CNS), as opportunists, become one of a major cause of hospital acquired infection (1).

Since *Staphylococcus* spp. has become resistant to many important antibiotics, the possibility that “older” compounds such as erythromycin and clindamycin still have ability to maintain the efficiency is very important to keep new resistance from developing. Over the past decade, an excessive and inappropriate use of antibiotics for human and animal treatment, as well as, animal feed supplements for growth promotion, has led to an increase in a number of staphylococci acquiring cross-resistance to macrolides, lincosamides, and streptogramins (MLS) antibiotics.

The mechanisms of resistance to MLS antibiotics are mainly related to the inhibition of protein synthesis. This can be mediated by several mechanisms: (a) ribosomal binding site modification (by methylation or mutation in the 23S rRNA gene) encoded by *erm* genes (*erm*A, *erm*B, *erm*C, *erm*Y, and *erm*F), (b) active efflux mediated by *msr*A/B gene, and (c) enzymatic modification of antibiotics (2).

The dimethylation of adenine A2058, at the N6 position which is located in the region of the peptidyl transferase loop in domain V of 23S rRNA in the 50S ribosomal subunit of bacteria, leads to cross-resistance between macrolides, lincosamides, and streptogramin group B (MLSb) (2). The family of genes responsible for this methylation is named *erm* (erythromycin ribosomal methylase) and now there are 21 different classes of identified *erm* genes (3). Since the erythromycin binding site on the 50S ribosome subunit overlaps the binding site of the newer MLSb, the modification by methylase(s) reduces the binding of all three classes of antibiotics, causing MLSb resistance phenotype (4). The expression of *erm* genes is manifested as either constitutive or inducible macrolides, lincosamides, and streptogramin B phenotype [constitutive resistance to macrolide–lincosamide–streptogramin B (cMLSb) or inducible resistance to macrolide–lincosamide–streptogramin B (iMLSb)]. In iMLSb phenotype, the bacteria produce inactive methylase mRNA, which becomes active only in the presence of a macrolide as an inducer. In the presence of inducer (erythromycin) a rearrangement of the mRNA occurs, allowing the methylase-coding sequence translation (5). In contrast, in bacteria showing cMLSb resistance phenotype, active methylase mRNA is produced in the absence of an inducer.

The active efflux of antibiotics is the second important mechanism of resistance in staphylococci. The *msr*A, *msr*B, and newly discovered *msr*C gene encode the ATP-dependent efflux pump (ABC), which determines resistance to 14-membered and 15-membered macrolides and streptogramin type B in *Staphylococcus* spp. (MSb resistance phenotype) (6). An active efflux ABC transporter-like transmembrane protein is encoded by *lsa* genes: *lsa*A and *lsa*C. It belongs to mechanism of resistance mediated by active efflux of antibiotics, causing the resistance to lincosamides and streptogramin type A phenotype (7).

In contrast to MLSb resistance phenotype, specific resistance to lincosamides and streptogramin type B (LSb) is mediated by enzymatic inactivation of the antibiotic. The most important enzymes that modify antibiotics are lincosamide nucleotidyl transferases encoded by *lnu* genes (formerly *lin*): *lnu*A and *lnu*B genes. In staphylococci, these enzymes are responsible for resistance to lincosamides and streptogramin type B phenotype (8).

Macrolides, lincosamides, and streptogramins resistance genes are responsible for resistance to MLS in community- and hospital-acquired *Staphylococcus* infections. Therefore, the purpose of the study was to analyze the distribution of the MLS resistance genes in *Staphylococcus* isolates and their distribution among community- and hospital-acquired isolates.

## Materials and Methods

### Bacterial Strains

A total of 1,643 staphylococcus isolates collected during 1-year period at the Center for Microbiology of Public Health Institute in Vranje were obtained from various clinical specimens including nasal and throat swabs, purulent discharge, and genital secretions, originating from both outpatient and inpatient populations. Multiple specimens from the same patient were avoided. The presence of the MLS resistance genes: *erm*A, *erm*B, *erm*C, *msr*A/B, *lnu*A, *lnu*B, and *lsa*A was determined in 179 staphylococcal isolates by end-point polymerase chain reaction (PCR). The bacterial DNA extraction and amplification of the specific resistance genes were performed at the Center for Microbiology Institute of Public Health in Kragujevac, Serbia.

The local ethics committee approved the study according to the Declaration of Helsinki (No. 01-5072/2013). The authors declare that informed consent was not required.

### Isolation and Identification

Isolation and identification of bacterial strains were performed using routine microbiological tests. The strains were identified to the species or genus level by the conventional microbiological methods.

### Determination of Resistance Phenotypes

All recovered isolates were tested by the double-disc diffusion method. The MLS resistance phenotypes: cMLSb, iMLSb, resistance to macrolide/macrolide–streptogramin B (M/MSb), and resistance to lincosamide–streptogramin A/streptogramin B (LSa/b) were determined by double-disc diffusion method according to Clinical and Laboratory Standards Institute recommendations (9). Erythromycin (15 µg) and clindamycin (2 µg) disks were placed at an edge-to-edge distance of 12 mm on inoculated Mueller–Hinton agar. Resistance to erythromycin and clindamycin indicates a constitutive MLSb resistance (cMLSb). The clindamycin diffusion zone which was blunted proximal to the erythromycin disk or showing D shape was considered as inducible type of resistance (iMLSb phenotype). Susceptibility to clindamycin and resistance to erythromycin defined the M/MSb phenotype. The isolates resistant to clindamycin and sensitive to erythromycin were defined as LSa/b phenotype.

### Isolation of Bacterial DNA

Bacterial DNA were extracted by PrepMan Ultra sample Preparation Reagent (Applied Biosystems, Inc.), according to the manufacturer’s guidelines. All extracted DNA samples were stored at −70°C prior to further analysis.

### Identification of Genes by Multiplex PCR

The sequences of the primers are presented in Table 1. PCR conditions for the primer sets have been as previously described by Rizzotti et al., Matsuoka et al., Lozano et al., and Singh and Murray in their studies (10–13). The final volume of each PCR reaction was 50 µl and contained 2 µl of genomic DNA, 1 µl of each primer (Invitrogen), 25 µl of Maxima^®^ Hot Start Green PCR Master Mix (Fermentas), and 21 µl of DEPC H_2_O. Positive and negative controls were included in each assay. The reactions were performed using the Sa Cycler-96 thermocycler (Sacace Biotechnologies S.r.l. Como, Italy), whereas the PCR products were detected by gel electrophoresis on the E-Gel iBase (Invitrogen) in 2% (w/v) agarose gel (E-Gel^®^ 2%, Invitrogen) and visualized on the Gel Doc XR System, including an ultraviolet light transilluminator (Bio-Rad) (14).

**Table 1:** Primer sequences and PCR fragment size of tested MLS resistance genes.

Gene	Primers sequence (5′–3′)	PCR fragment size (bp)	Reference
*erm*A	F: TCTAAAAAGCATGTAAAAGAA	645	(10)
	R: CTTCGATAGTTTATTAATATTAG		
*erm*B	F: GAAAAGTACTCAACCAAATA	639	(10)
	R: AGTAACGGTACTTAAATTGTTTA		
*erm*C	F: TCAAAACATAATATAGATAAA	642	(10)
	R: GCTAATATTGTTTAAATCGTCAAT		
*msr*A	F: GGCACAATAAGAGTGTTTAAAGG	940	(11)
	R: AAGTTATATCATGAATAGATTGTCCTGTT		
*msr*B	F: TATGATATCCATAATAATTATCCAATC	595	(11)
	R: AAGTTATATCATGAATAGATTGTCCTGTT		
*lnu*A	F: GGTGGCTGGGGGGTAGATGTATTAACTGG	323	(12)
	R: GCTTCTTTTGAAATACATGGTATTTTTCGATC		
*lnu*B	F: CCTACCTATTGTTTGTGGAA	925	(12)
	R: ATAACGTTACTCTCCTATTC		
*lsa*A	F: GGCAATCGCTTGTGTTTTAGCG	1,200	(13)
	R: GTGAATCCCATGATGTTGATACC		

*MLS, macrolides, lincosamides, and streptogramins; PCR, polymerase chain reaction*.

### Statistics

Fisher’s exact test is used to compare two proportions at a significance level of *p* < 0.05. The results of the experiments are presented in tables and figures.

## Results

Among 1,643 examined isolates of staphylococci, 944 were identified as *S. aureus*. The rest of 699 isolates were characterized as CNS. Those isolates were further characterized by double-disc diffusion method to determine the MLS resistance phenotype. However, the largest number of staphylococcal isolates showed sensitivity to both macrolide and lincosamide (39.1%). Among staphylococcal isolates the most common resistance phenotype was iMLSb (33.4%), the second most prevalent was M/MSb (17.6%). cMLSb phenotype was detected in only 8.9% of staphylococcal isolates, and the rarest detected resistance phenotype was LSa/b (1%).

Distribution of MLS resistance phenotypes by origin of the staphylococcal isolate (community acquired versus nosocomial) is presented in Table 2. The most frequent MLS phenotype among staphylococci isolates, both community-acquired and hospital-acquired, was iMLSb. There were no statistically significant differences between community- and hospital-acquired staphylococci isolates showing iMLSb, cMLSb, LSa/b phenotype, and sensitivity to both erythromycin and clindamycin (*p* > 0.05). However, there was statistically significantly higher number of hospital-acquired staphylococcal isolates showing M/MSb phenotype (*p* < 0.05) (Table 2).

**Table 2:** Distribution of MLS resistance phenotypes by origin of the staphylococcal isolate (community-acquired versus hospital-acquired); *p*-value < 0.05 is considered as statistically significant.

	*Staphylococcus* spp., *n* (%)		
	Community-acquired	Hospital-acquired	*p*-Value
Er/Cli S	546 (39.9)	97 (35.1)	0.16
cMLSb	128 (9.3)	19 (6.8)	0.20
M/MSb	**218 (15.9)**	**71 (25.7)**	**0.0001**
iMLSb	461 (33.7)	87 (31.5)	0.52
LSa/b	14 (1.0)	2 (0.7)	1.0
Total	1,367 (100.0)	276 (100.0)	

*Er/Cli S, susceptibility to erythromycin and clindamycin; cMLSb, constitutive resistance to macrolide–lincosamide–streptogramin B; M/MSb, resistance to macrolide/macrolide–streptogramin B; iMLSb, inducible resistance to macrolide–lincosamide–streptogramin B; LSa/b, resistance to lincosamide–streptogramin A/streptogramin B*.

One hundred and 79 strains of staphylococcus isolates showing different MLS phenotypes were selected for further analysis. End-point PCR was performed to detect clinically relevant MLS resistance genes. The most commonly detected MLS resistance genes among staphylococci isolates were *msr*A/B, followed by *erm* genes: *erm*C, *erm*B, and *erm*A. In 9.8% of staphylococcal isolates showing iMLSb, M/MSb, or LSa/b resistance phenotype, MLS resistance genes have not been found.

The observed MLS phenotypes of resistance were the most frequently determined by a single gene, and those genes mostly were *msr*A/B and *erm*C. However, these phenotypes were also determined by combinations of two or more resistance genes and the most common detected gene combinations were *erm*C + *msr*A/B, *erm*B + *lsa*A, and *erm*B + *msr*A/B.

*erm* A and *erm*C genes were the most common in methicillin-sensitive *S. aureus* (MSSA) isolates, while *erm*B gene was the most frequent among methicillin-resistant coagulase-negative staphylococci (MRCNS). The greatest presence of *lnu*A genes was detected in methicillin-sensitive coagulase-negative staphylococci (MSCNS), whereas *lsa*A gene was, as a single case, detected only in *S. aureus* isolates. The highest percentage of msrA/B gene was identified in MSCNS. The combinations of two or more resistance genes were the most common in methicillin-resistant staphylococci isolates (Table S1 in Supplementary Material).

**Figure 1:**
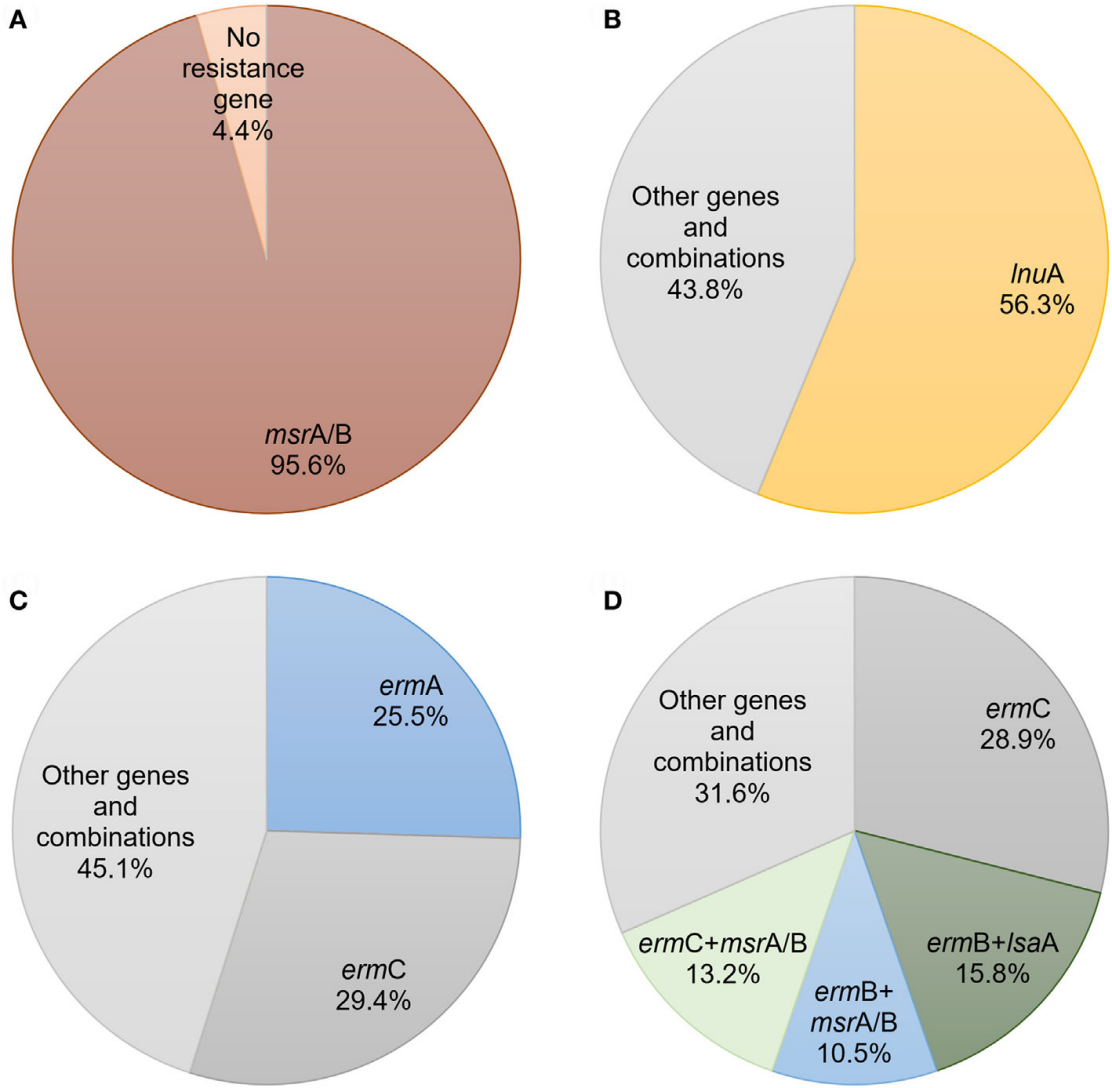
The prevalence and expression of macrolides, lincosamides, and streptogramins resistance genes and their combinations among different phenotypes of staphylococcal isolates: **(A)** resistance to macrolide/macrolide–streptogramin B; **(B)** resistance to lincosamide–streptogramin A/streptogramin B; **(C)** inducible resistance to macrolide–lincosamide–streptogramin B; **(D)** constitutive resistance to macrolide–lincosamide–streptogramin B.

M/MSb resistance phenotype was mostly determined by *msr*A/B (95.6%) (Figure 1A), while LSa/b phenotype was mostly determined by *lnu*A gene (56.3%) (Figure 1B). *erm*C (29.4%) and *erm*A (25.5%) were the most prevalent genes determined as a single gene iMLSb phenotype (Figure 1C). However, *erm*C (28.9%) was the most common gene in staphylococcal isolates with cMLSb-resistant phenotype. This phenotype was also characterized by the presence of great number of combinations with two or more resistance genes. The most common gene combinations in these isolates were *erm*B + *lsa*A (15.8%), *erm*C + *msr*A/B (13.2%), and *erm*B + *msr*A/B (10.5%) (Figure 1D) (Table S2 in Supplementary Material). This trend was especially observed in methicillin-resistant *S. aureus* (MRSA) (58.3%) and MRCNS (90.9%) strains. Combinations of genes predominantly determined cMLSb phenotype in methicillin-resistant staphylococci. In accordance with this observation, in one MRCNS strain the combination of four genes (*erm*B + *lnu*A + *lnu*B + *lsa*A) has been detected.

Distribution of MLS resistance genes and their combinations by origin of the staphylococcal isolate are shown in Table 3. The *msr*A/B gene was statistically significantly more common in hospital-acquired staphylococci isolates than in community-acquired staphylococci strains (*p* < 0.05). There are no statistically significant differences between percentage of staphylococci harboring the rest of MLS resistance genes acquired in community and hospital settings (*p* > 0.05).

**Table 3:** Distribution of macrolides, lincosamides, and streptogramins resistance genes by origin of the staphylococcal isolate (community-acquired versus hospital-acquired); *p*-value < 0.05 is considered as statistically significant.

	*Staphylococcus* spp., *n* (%)		
	Community-acquired	Hospital-acquired	*p*-Value
*erm*A	13 (8.6)	1 (3.4)	0.47
*erm*B	4 (2.6)	2 (6.9)	0.25
*erm*C	24 (16.0)	2 (6.9)	0.26
*lnu*A	7 (4.6)	3 (10.3)	0.21
*lsa*A	2 (1.3)	0	1.00
*msr*A/B	**34 (22.6)**	**13 (44.8)**	**0.02**
*erm*A + *erm*C	1 (0.6)	0	1.00
*erm*A + *msr*A/B	2 (1.3)	0	1.0
*erm*B + *erm*C	1 (0.6)	0	1.0
*erm*B + *lsa*A	5 (3.3)	1 (3.4)	1.0
*erm*B + *msr*A/B	4 (2.6)	0	1.0
*erm*C + *lsa*A	1 (0.6)	0	1.0
*erm*C + *msr*A/B	7 (4.6)	1 (3.4)	1.0
*lnu*A + *lnu*B	1 (0.6)	0	1.0
*msr*A/B + *lsa*A	1 (0.6)	0	1.0
*erm*B + *msr*A/B + *lsa*A	0	1 (3.4)	0.16
*erm*C + *msr*A/B + *lnu*A	1 (0.6)	0	1.0
*erm*B + *lnu*A + *lnu*B + *lsa*A	1 (0.6)	0	1.0
No resistance gene	41 (27.3)	5 (17.2)	0.35
Total	150 (100)	29 (100)	

## Discussion

Soon after penicillin was introduced, *S. aureus* strains resistant to penicillin had been found. These penicillin-resistant *S. aureus* strains first became prevalent in health-care settings but then, these strains have also been spread into the community. Rapidly after introduction of newer antibiotics such as methicillin, erythromycin, and clindamycin, *S. aureus* and some other staphylococci species have become resistant to those and other antibiotics. Finally, multidrug-resistant *Staphylococcus* strains have evolved, often causing infections with a fatal outcome (15). Multidrug-resistant *S. aureus* and CNS have become a common cause of both hospital- and community-acquired infection. The necessity in phenotypic and genotypic tests for discovering resistance in clinically relevant staphylococci has become clearer with the occurrence of strains having borderline minimum inhibitory concentrations of antibiotics. Identifying MLS resistance phenotype is very important, because iMLSb phenotype under intensive antibiotic selective pressure converts into cMLSb phenotype and may lead to treatment failure in patients with serious staphylococcal infection (16).

In our study, the greatest number of staphylococcal isolates showed sensitivity to both macrolide and lincosamide and the most frequent phenotype of resistance was iMLSb, while cMLSb phenotype was almost the rarest MLS resistance phenotype. This pattern of MLS resistance was the same among community- and hospital-associated staphylococcal isolates. Different distribution of MLS resistance phenotype among methicillin-resistant staphylococci isolates in India has been reported by Zachariah et al. (16). They found that the most prevalent phenotype was M/MSb, and the second most prevalent phenotype was iMLSb. In contrast, Hamilton-Miller and Shah, Fokas et al. have found that iMLSb phenotype was more prevalent in relation to the other phenotypes (17, 18), data similar to our study. The distribution of MLS resistance phenotypes may vary depending on geographic area and even the type of patient. This difference occurs because of various prescription and consumption rates of macrolides and lincosamides in different geographical regions and even institutions in the same region because of different origins of the isolates (hospital- versus community-acquired) (19). In our study, we found that M/MSb phenotype was significantly higher in inpatient than outpatient isolates (*p* < 0.05). Similarly, we have detected that *msr*A gene determining M/MSb phenotype more frequently in hospital-acquired than in community-acquired staphylococcal isolates (*p* < 0.05). This finding suggests importance of phenotypic differentiation of truly clindamycin sensitive from false clindamycin-sensitive staphylococci (iMLSb), especially for staphylococci isolates from hospital environments. In contrast, Lall and Sahni suggested a higher prevalence of iMLSb in health care-associated *Staphylococcus* than community associated (86.5 versus 13.4%, respectively) (20). Yet, the implication of their study is the same as ours.

The molecular analysis identified the *msr*A gene, encoding active efflux pumps in staphylococci bacterial cells, as the most frequent MLS resistance gene. More than half of the macrolide-resistant isolates of staphylococci harbored the *msr*A gene either alone or in combination with *erm* genes. Recent studies demonstrated similar results (21, 22). The *erm*C gene was the most common among all *erm* genes in both *S. aureus* and CNS exhibiting cMLSb or iMLSb phenotype. Similar data were reported by Juda et al. (23). In Brazil, Coutinho et al. (24) reported low frequency of the *erm*B gene, data which did not differ from our study. However, there was only a small number of staphylococcal isolates with the unusual LSa/b phenotype harboring *lnu*A and *lsa*A genes, in this study. These results are in line with the studies of Singh and Murray and Deng et al. (13, 25). The most common detected gene combinations in our study were *erm*C + *msr*A/B, *erm*B + *lsa*A, and *erm*B + *msr*A/B. As expected, we identified the greatest number of resistance gene combinations in methicillin-resistant staphylococci isolates. Likewise, the simultaneous presence of two or more MLS resistance genes in the same staphylococcal isolate has been reported previously for hospital-acquired MRSA and MRCNS isolates in Argentina, USA, and Poland, respectively (26–28). We proposed that the majority of isolates with simultaneous presence of two or more MLS resistance genes would have been found among strains isolated from inpatients. Regardless of our expectation, there were no significant differences in number of staphylococcal isolates with MLS gene combinations between hospital- and community-acquired strains (*p* > 0.05). 9.8% of our isolates showing iMLSb, M/MSb, or LSa/b resistance phenotype had no MLS resistance gene. Similar finding has been reported in other studies (19, 29).

In the present study, the highest percentage of *msr*A/B gene was identified in MSCNS. Macrolide resistance due to *msr*A was more prevalent in CNS than in *S. aureus*. Similar data were observed in one study (30), whereas in other studies, the presence of *msr*A/B genes has been reported in different rates (31–33). In our study, *erm*A and *erm*C genes were most common in MSSA isolates. In contrast, Westh et al. (34) detected that *erm*A gene is most common in MRSA isolates, whereas *erm*C gene was mostly found among their MRCNS isolates. We found *erm*B gene mostly among MRCNS, whereas in the study conducted by Bouchami et al. *erm*B has not been detected in staphylococci (35). In our study, *lnu*A gene was the most commonly detected in MSCNS and similar, almost the same, results were found in study by Lina et al. (36). For the first time, we detected *lsaA* gene as a single gene in *S. aureus* isolates. *lsa*A gene in enterococci encodes a protein, similar to ABC transporters, which export antimicrobials belonging to the MLS family (37). The *lsa*A gene as an intrinsic gene of *Enterococcus faecalis* showed a high degree of similarity to a novel gene encoding the ABC transporter (*lsa*E gene), which has been already detected in staphylococci. Transfer of resistance from enterococci to *S. aureus* has also been reported to occur for the tetracycline resistance gene *tet*L and the trimethoprim resistance gene *dfr*K (38). Yet, the *lsa*A gene, originating from *Enterococcus* spp. (39), has never been reported before for *S. aureus*.

In our study, M/MSb resistance phenotype in staphylococci isolates was mostly determined by *msr*A/B, while LSa/b phenotype was mostly determined by *lnu*A gene. *erm*C and *erm*A were the most prevalent single genes determining iMLSb phenotype. In other studies, the presence of *erm*C and *erm*A genes has been reported with different rates (19, 29, 33, 40, 41). However, *erm*C gene was the most common gene in staphylococcal isolates with cMLSb-resistant phenotype. This result is in accordance to the previous report (42). cMLSb phenotype was also characterized by the presence of great number of gene combinations: *erm*B + *lsa*A, *erm*C + *msr*A/B, and *erm*B + *msr*A/B. This trend was particularly observed in MRSA and MRCNS strains, where these combinations of genes predominantly determined cMLSb phenotype. In accordance with this observation, in one MRCNS strain the combination of four genes (*erm*B + *lnu*A + *lnu*B + *lsa*A) has been detected. Hosseini et al. have come across pattern of similar kind (43).

After all, according to all investigated staphylococci, the iMLSb was the most frequently occurring phenotype. The most frequently isolated MLS resistance genes among staphylococci were *msr*A/B and *erm*C. M/MSb (*msr*A/B), LSa/b (*lnu*A), and iMLSb (*erm*A/C) were dominantly determined with a single gene. cMLSb phenotype was mostly determined by *erm*C and combinations of genes (*erm*C + *msr*A/B and *msr*A/B + *lsa*A). The M/MSb phenotype and *msr*A/B gene that determine this phenotype were significantly more frequent in staphylococci acquired in hospital than in community. Based on these results, clindamycin can be used for empiric antimicrobial therapy for infections such as skin and soft tissue infections in inpatients, before the individual’s laboratory results of *in vitro* antibiotic susceptibility testing are available.

However, the iMLSb phenotype was the most common in our study, either among community- or hospital-acquired *Staphylococcus* isolates. Therefore, the big concern remains for patients with severe staphylococcal infection treated with clindamycin, they could be at risk of unsuccessful treatment and poor outcome if staphylococcal isolates showed inducible clindamycin resistance (44). The simple test like D-test on staphylococcal strains can separate the isolates with genetic mechanism for the development of clindamycin resistance during therapy from those that are truly susceptible to clindamycin. If we do not perform routinely D-test, all strains of staphylococci including those with iMLSb resistance phenotype will be reported as clindamycin sensitive. On the contrary, if all erythromycin-resistant staphylococcal strains are declared as a resistant to clindamycin, a safe and effective antibiotic will be omitted from the treatment in patients infected with isolates carrying an active efflux mechanism that confers only resistance to macrolides (35). In our study, we reported that 56.7% actually (M/MSb and susceptibility to erythromycin and clindamycin) and 33.4% falsely (iMLSb) clindamycin-sensitive *Staphylococcus* spp. strains have led to such a small percentage of clindamycin resistance 9.9% (cMLSb and LSa/b) instead of 49.0% (iMLSb and cMLSb and LSa/b) among staphylococcal isolates. Therefore, it is necessary to monitor the prevalence of iMLS phenotype, especially in areas where the occurrence of methicillin-resistant staphylococcal strains resulted in empirical use of macrolides and lincosamides for the treatment of staphylococcal infections. The prevalence of iMLSb phenotypes may change over time, so it is necessary to perform sporadically survey of MLS resistance phenotypes, particularly where the D-test is not performed routinely.

## Author Contributions

MM contributed to the conception, design, and drafting of the study, data acquisition and processing, and writing of the manuscript. JČ contributed to the conception of the study, data acquisition, and corrected the manuscript. DV contributed to the design of the study, data analysis and interpretation, and corrected the manuscript. MŠ contributed to the conception of the study, data analysis and interpretation, and corrected the manuscript. SM contributed to the conception of the study, data analysis and interpretation, and drafting of the study. MV contributed to the conception and design of the study, data analysis and interpretation, and corrected the manuscript. DB contributed to the conception and design of the study, guided in data acquisition, analysis, and interpretation, and corrected the manuscript.

## Conflict of Interest Statement

The authors declare that the research was conducted in the absence of any commercial or financial relationships that could be construed as a potential conflict of interest.

## Abbreviations

MLS, macrolides, lincosamides, and streptogramins; Er/Cli S, susceptibility to erythromycin and clindamycin; cMLSb, constitutive resistance to macrolide–lincosamide–streptogramin B; M/MSb, resistance to macrolide/macrolide–streptogramin B; iMLSb, inducible resistance to macrolide–lincosamide–streptogramin B; LSa/b, resistance to lincosamide–streptogramin A/streptogramin B; MSSA, methicillin-sensitive *S. aureus*; MRSA, methicillin-resistant *S. aureus*; MSCNS, methicillin-sensitive coagulase-negative staphyloccoci; MRCNS, methicillin-resistant coagulase-negative staphylococci; PCR, polymerase chain reaction; CLSI, Clinical and Laboratory Standards Institute.
