# Supplementary material for: Prevalence of Genotypes That Determine Resistance of Staphylococci to Macrolides and Lincosamides in Serbia
Source: Front Public Health. 2017 Aug 28;5:200. doi: 10.3389/fpubh.2017.00200 (PMC5581325; doi:10.3389/fpubh.2017.00200)
Supplement: Supplementary file 1 [file table_1.docx]

Supplementary Material

Prevalence of genotypes that determine resistance of staphylococci to macrolides and lincosamides in Serbia

Milena Mišić^1^, Jelena Čukić^2^, Dejan Vidanović^3^, Milanko Šekler^3^, Sanja Matić^4^, Mihailo Vukašinović^4^, Dejan Baskić^2,5*^

*** Correspondence:** MD. PhD. Dejan Baskić: dejan.baskic@gmail.com

**Supplementary Table 1.** Distribution of macrolide, lincosamide and streptogramin resistance genes, and their combinations in different staphylococci groups.

MSSA, methicillin-susceptible *S. aureus*; MRSA, methicillin-resistant *S. aureus*; MSCNS, methicillin-susceptible coagulase-negative staphylococcoci; MRCNS, methicillin-resistant coagulase-negative staphylococci.

|  | **MSSA n (%)** | **MRSA n (%)** | **MSCNS n (%)** | **MRCNS n (%)** | **Total n (%)** |
| --- | --- | --- | --- | --- | --- |
| ***erm*A** | 8 (16.7) | 3 (6.7) | 2 (4) | 1 (2.8) | 14 (7.8) |
| ***erm*B** |  | 2 (4.4) | 1 (2) | 3 (8.3) | 6 (3.4) |
| ***erm*C** | 8 (16.6) | 7 (15.6) | 6 (12) | 5 (13.9) | 26 (14.5) |
| ***lnu*A** | 1 (2.1) | 3 (6.6) | 6 (12) |  | 10 (5.6) |
| ***lsa*A** | 1 (2.1) | 1 (2.2) |  |  | 2 (1.1) |
| ***msr*A/B** | 14 (29.2) | 11 (24.4) | 15 (30) | 7 (19.4) | 47 (26.3) |
| ***erm*A+*erm*C** | 1 (2.1) |  |  |  | 1 (0.6) |
| ***erm*A+*msr*A/B** | 1 (2.1) | 1 (2.2) |  |  | 2 (1.1) |
| ***erm*B+*erm*C** |  |  |  | 1 (2.8) | 1 (0.6) |
| ***erm*B+*lsa*A** |  |  | 1 (2) | 5 (13.9) | 6 (3.4) |
| ***erm*B+*msr*A/B** |  | 3 (6.7) |  | 1 (2.8) | 4 (2.2) |
| ***erm*C+*lsa*A** | 1 (2.1) |  |  |  | 1 (0.6) |
| ***erm*C+*msr*A/B** |  | 2 (4.4) | 2 (4) | 4 (11.2) | 8 (4.5) |
| ***lnu*A+*lnu*B** |  |  | 1 (2) |  | 1 (0.6) |
| ***msr*A/B+*lsa*A** |  | 1 (2.2) |  |  | 1 (0.6) |
| ***erm*B+*msr*A/B+*lsa*A** |  | 1 (2.2) |  |  | 1 (0.6) |
| ***erm*C+*msr*A/B+*lnu*A** | 1 (2.1) |  |  |  | 1 (0.6) |
| ***erm*B+*lnu*A+*lnu*B+*lsa*A** |  |  |  | 1 (2.8) | 1 (0.6) |
| **No resistance gene** | 12 (25) | 10 (22.1) | 16 (32) | 8 (22.2) | 46 (25.7) |
| **Total** | 48 (100) | 45 (100) | 50 (100) | 36 (100) | 179 (100) |
