# Supplementary material for: Prevalence of Genotypes That Determine Resistance of Staphylococci to Macrolides and Lincosamides in Serbia
Source: Front Public Health. 2017 Aug 28;5:200. doi: 10.3389/fpubh.2017.00200 (PMC5581325; doi:10.3389/fpubh.2017.00200)
Supplement: Supplementary file 2 [file table_2.docx]

Supplementary Material

Prevalence of genotypes that determine resistance of staphylococci to macrolides and lincosamides in Serbia

Milena Mišić^1^, Jelena Čukić^2^, Dejan Vidanović^3^, Milanko Šekler^3^, Sanja Matić^4^, Mihailo Vukašinović^4^, Dejan Baskić^2,5*^

*** Correspondence:** MD. PhD. Dejan Baskić: dejan.baskic@gmail.com

**Supplementary Table 2.** The prevalence of MLS resistance genes and their combinations among different MLS phenotypes of staphylococcal isolates.

Er/Cli S, susceptibility to erythromycin and clindamycin; cMLSb, constitutive resistance to macrolide-lincosamide-streptogramin B; M/MSb, resistance to macrolide/macrolide-streptogramin B; iMLSb, inducible resistance to macrolide-lincosamide-streptogramin B; LSa/b, resistance to lincosamide-streptogramin A/streptogramin B.

|  | ***Staphylococcus* spp.** | | | | | |
| --- | --- | --- | --- | --- | --- | --- |
|  | **Er/Cli S** | **cMLSb** | **M/MSb** | **iMLSb** | **LSa/b** | **Total** |
| ***erm*A** |  | 1 (2.6) |  | 13 (25.5) |  | 14 (7.8) |
| ***erm*B** |  | 2 (5.3) |  | 4 (7.8) |  | 6 (3.4) |
| ***erm*C** |  | 11 (28.9) |  | 15 (29.4) |  | 26 (14.5) |
| ***lnu*A** |  |  |  | 1 (2) | 9 (56.3) | 10 (5.6) |
| ***lsa*A** |  | 1 (2.6) |  |  | 1 (6.3) | 2 (1.1) |
| ***msr*A/B** | 1 (3.4) | 1 (2.6) | 43 (95.6) | 1 (2) | 1 (6.3) | 47 (26.3) |
| ***erm*A+*erm*C** |  |  |  | 1 (2) |  | 1 (0.6) |
| ***erm*A+*msr*A/B** |  | 1 (2.6) |  | 1 (2) |  | 2 (1.1) |
| ***erm*B+*erm*C** |  | 1 (2.6) |  |  |  | 1 (0.6) |
| ***erm*B+*lsa*A** |  | 6 (15.8) |  |  |  | 6 (3.4) |
| ***erm*B+*msr*A/B** |  | 4 (10.5) |  |  |  | 4 (2.2) |
| ***erm*C+*lsa*A** |  | 1 (2.6) |  |  |  | 1 (0.6) |
| ***erm*C+*msr*A/B** |  | 5 (13.2) |  | 3 (5.9) |  | 8 (4.5) |
| ***lnu*A+*lnu*B** |  |  |  |  | 1 (6.3) | 1 (0.6) |
| ***msr*A/B+*lsa*A** |  | 1 (2.6) |  |  |  | 1 (0.6) |
| ***erm*B+*msr*A/B+*lsa*A** |  | 1 (2.6) |  |  |  | 1 (0.6) |
| ***erm*C+*msr*A/B+*lnu*A** |  | 1 (2.6) |  |  |  | 1 (0.6) |
| ***erm*B+*lnu*A+*lnu*B+*lsa*A** |  | 1 (2.6) |  |  |  | 1 (0.6) |
| **No resistance gene** | 28 (96.6) |  | 2 (4.4) | 12 (23.5) | 4 (25) | 46 (25.7) |
| **Total** | 29 (100) | 38 (100) | 45 (100) | 51 (100) | 16 (100) | 179 (100) |
